# High triglyceride-glucose index predicts cardiovascular events in patients with coronary bifurcation lesions: a large-scale cohort study

**DOI:** 10.1186/s12933-023-02016-x

**Published:** 2023-10-27

**Authors:** Jining He, Sheng Yuan, Chenxi Song, Yanjun Song, Xiaohui Bian, Guofeng Gao, Kefei Dou

**Affiliations:** 1 State Key Laboratory of Cardiovascular Disease, Beijing, China; 2 Cardiometabolic Medicine Center, National Clinical Research Center for Cardiovascular Diseases, State Key Laboratory of Cardiovascular Disease, Fuwai Hospital, National Center for Cardiovascular Diseases, Chinese Academy of Medical Sciences and Peking Union Medical College, A 167 Beilishi Rd, Xicheng District, Beijing, 100037 China; 3 Department of Cardiology, Fuwai Hospital, National Center for Cardiovascular Diseases, Chinese Academy of Medical Sciences and Peking Union Medical College, A 167 Beilishi Rd, Xicheng District, Beijing, 100037 China; 4 National Clinical Research Center for Cardiovascular Diseases, Beijing, China

**Keywords:** Coronary bifurcation lesions, Diabetes Mellitus, Triglyceride-glucose index, Prognosis

## Abstract

**Background:**

Coronary bifurcation lesion, as a complex coronary lesion, is associated with higher risk of long-term poor prognosis than non-bifurcation lesions. The triglyceride-glucose (TyG) index has been shown to predict cardiovascular (CV) events in patients with coronary artery disease (CAD). However, the prognostic value of the TyG index in patients with bifurcation lesions who are at high risk of CV events remains undetermined. Therefore, this study aimed to investigate the association between the TyG index and CV events in patients with bifurcation lesions.

**Methods:**

A total of 4530 consecutive patients with angiography-proven CAD and bifurcation lesions were included in this study from January 2017 to December 2018. The TyG index was calculated as Ln [fasting triglyceride (mg/dL) × fasting plasma glucose (mg/dL)/2]. Patients were assigned into 3 groups according to TyG tertiles (T) (T1: <8.633; T2: 8.633–9.096 and T3: ≥9.096). The primary endpoint was CV events, including CV death, nonfatal myocardial infarction and nonfatal stroke at 3-year follow-up. Restricted cubic spline (RCS) analysis, Kaplan-Meier curves and Cox proportional hazard models were used to investigate the associations between the TyG index and study endpoints.

**Results:**

During a median follow-up of 3.1 years, 141 (3.1%) CV events occurred. RCS analysis demonstrated a linear relationship between the TyG index and events after adjusting for age and male sex (non-linear *P* = 0.262). After multivariable adjustments, elevated TyG index (both T2 and T3) was significantly associated with the risk of CV events (hazard ratio [HR], 1.68; 95% confidence interval [CI],1.06–2.65; HR, 2.10; 95%CI, 1.28–3.47, respectively). When study patients were further stratified according to glycemic status, higher TyG index was associated with significantly higher risk of CV events in diabetic patients after adjusting for confounding factors (T3 vs. T1; HR, 2.68; 95%CI, 1.17–6.11). In addition, subgroup analysis revealed consistent associations of the TyG index with 3-year CV events across various subgroups. Furthermore, adding the TyG index to the original model significantly improved the predictive performance.

**Conclusions:**

High TyG index was associated with CV events in patients with bifurcation lesions, suggesting the TyG index could help in risk stratification and prognosis in this population.

**Supplementary Information:**

The online version contains supplementary material available at 10.1186/s12933-023-02016-x.

## Background

Coronary bifurcation lesions, accounting for about 15–20% of coronary artery disease (CAD), are one of the complex coronary lesions [1, 2]. In the interventional practice, bifurcation lesions are associated with lower procedural success rates and worse clinical outcomes [3, 4]. Meanwhile, the bifurcation lesions are also associated with higher risk of stent thrombosis [5]. A previous post hoc subgroup analysis of The SYNTAX (Synergy Between PCI With Taxus and Cardiac Surgery) Extended Survival (SYNTAXES) study has showed that patients with bifurcation lesions had higher 10-year all-cause mortality after intervention than those with non-bifurcation lesions [3]. Therefore, approaches that could identify high-risk patients for cardiovascular (CV) events among those with bifurcation lesions are warranted to help in risk stratification and therapeutic management.

Increasing evidence have shown that insulin resistance (IR) and its related conditions are not only a hallmark of diabetes mellitus (DM) but also a risk factor for CV diseases (CVD) [6]. Arguably, the gold standards of IR diagnosis are euglycemic insulin clamp and intravenous glucose tolerance testing. Nonetheless, these methods have not been applied in clinical practice because of invasiveness and high cost. In addition, the homeostasis model assessment estimated insulin resistance (HOMA-IR) index, a means for detecting β-cell function and IR, is currently widely used, but it has limited practical value in individuals receiving insulin treatment or not having functional beta cells [7]. In recent years, the triglyceride-glucose (TyG) index has been developed and favored by many investigators as a simple and reliable surrogate to assess IR in individuals with or without DM [6]. Previous studies have suggested that increased TyG index levels were not only associated with the incidence of CVD, such as CAD, coronary artery calcification, carotid artery atherosclerosis, and metabolic related diseases, but also can predict prognosis in patients with established CVD [6, 8–10]. However, the prognostic value of the TyG index in patients with bifurcation lesion who were at higher risk of adverse clinical events remained unclear.

Therefore, this study aimed to determine the association between the TyG index and CV events in CAD patents with bifurcation lesions.

## Methods

### Study design and population

**Fig. 1:**
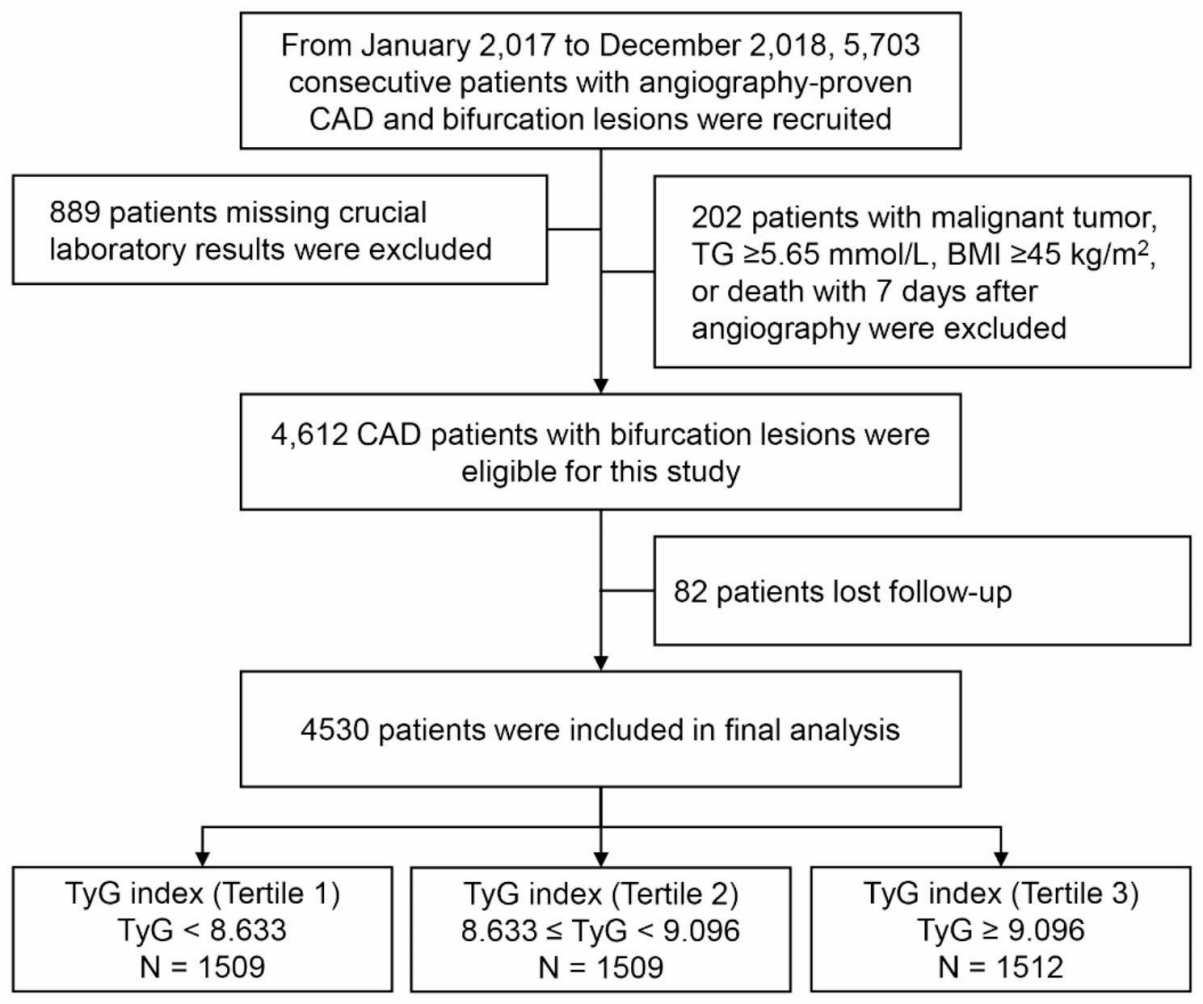
Study flowchart. BMI, body mass index; CAD, coronary artery disease; TG, triglyceride; TyG, triglyceride-glucose

This was a single-center prospective cohort study. From January 2017 to December 2018, a total of 4530 consecutive CAD patients with bifurcation lesions were enrolled at Fuwai Hospital, Chinese Academy of Medical Sciences. The bifurcation lesions referred to a coronary artery narrowing occurring adjacent to, and/or involving the origin of a significant side branch [11]. The exclusion criterion included incomplete data for calculating the TyG index, triglyceride (TG) ≥ 5.65 mmol/L, body mass index (BMI) ≥ 45 mmol/L and death within 7 days after coronary angiography (CAG), and loss of follow-up. Finally, a total of 4530 patients with bifurcation lesions were included in this study. Detailed recruitment process is shown in Fig. 1. The TyG index was calculated using the reported equation: Ln (fasting triglyceride [mg/dL] × FBG [mg/dL] / 2) [3]. Patients were classified into 3 groups according to the TyG index tertiles: T1 (TyG < 8.633), T2 (8.633 ≤ TyG < 9.096), and T3 (TyG ≥ 9.096).

This study was conducted in compliance with the Declaration of Helsinki. The study protocol was approved by Fuwai Hospital’s Institutional Review Board. All patients provided written informed consent before enrollment.

### Study procedures and data collection

Patients were followed up at 6-month intervals after discharge until December 31, 2021. Endpoint data were gathered by trained investigators through telephone interviews with structured questionnaires, and/or clinical visits records. All treatments and procedures for patients were carried out in accordance with the recommendations of the guidelines and the cardiologist’s discretion. Two trained interventional cardiologists independently collected angiographic and procedural data from catheter laboratory records. Using standardized questionnaires, independent research personnel collected demographic and clinical data prospectively. Severe calcification was defined as readily apparent radiopacity within the vascular wall without cardiac motion before contrast injection, generally compromising both sides of the arterial lumen [12].

### Laboratory measurements

On admission, venous blood samples were drawn from each patient after at least 12-hour fasting, and analyzed in the clinical chemistry department of Fuwai Hospital. An automated biochemical analyzer (Hitachi 7150, Tokyo, Japan) was used to measure the concentrations of total cholesterol (TC), low-density lipoprotein cholesterol (LDL-C), high-density lipoprotein cholesterol (HDL-C), TG, fasting blood glucose (FBG), serum creatinine, and lipoprotein(a) [Lp(a)] with an enzymatic assay. Tosoh Automated Glycohemoglobin Analyzer (HLC-723G8, Tokyo, Japan) was used to quantify glycosylated hemoglobin A1c (HbA1c) [13, 14].

### Study endpoints and definitions

The primary endpoint was defined as 3-year CV events defined as a composite of CV death, non-fatal myocardial infarction (MI), and non-fatal stroke. The second endpoint was major adverse cardiovascular events (MACEs) defined as a composite of CV death and non-fatal MI at 3-year follow-up. Unless a clear non-cardiovascular reason could be proven, all deaths were deemed CV related. According to the third universal definition of MI, clinical and laboratory criteria were used to determine the diagnosis [2]. A new focal neurological deficit lasting more than 24 h that is established by neurologists using imaging data is referred to as a stroke. All events were confirmed and judged by two independent professional clinicians who were not aware of the study, and any disagreements were settled by consulting a third experienced clinician.

Glycemic states were determined according to American Diabetes Association criterion [4]. DM was defined as previous physician diagnosis of DM or receiving hypoglycemic drugs treatment, or FBG ≥ 126 mg/dL (7.0 mmol/L), or glycated hemoglobin A1c (HbA1c) levels ≥ 6.5%, or 2-hour blood glucose of oral glucose tolerance test (OGTT) ≥ 200 mg/dL (11.1 mmol/L), or use of insulin or oral hypoglycemic medication. Patients who met one of the following criteria were diagnosed as prediabetes: impaired fasting glucose (IFG) [FBG: 110–125 mg/dL (6.1–6.9 mmol/L)]; impaired glucose tolerance (IGT) [OGTT 2-hour glucose value ≥ 140 mg/dL (7.8 mmol/L) but < 200 mg/dL (11.1 mmol/L) and FBG < 126 (7.0 mmol/L)]; HbA1c 5.7–6.4%. Patients without DM or prediabetes were considered as normal glucose tolerance (NGT). Hypertension was diagnosed when the blood pressure was measured more than twice on separate days and the systolic blood pressure ≥ 140 mmHg on both days and/or diastolic blood pressure ≥ 90 mmHg on both days, or self-reported physician diagnosis of hypertension, or use of antihypertensive drugs.

### Statistical analysis

Continuous variables were summarized using the mean ± standard deviation (SD), whereas categorical variables were presented as frequency and percentage. Student’s t-test, Mann-Whitney U test or One-way ANOVA was used to compare continuous variables between groups, as appropriate, whereas the Chi-square test or Fisher’s exact test was employed to compare categorical variables, as appropriate.

The incidence of CV events and MACEs in different groups was depicted using Kaplan-Meier survival curves and compared using the Log-rank test. Spearman correlation analysis was performed to evaluate the correlation between the TyG index and clinical risk factors. Restricted cubic spline (RCS) was utilized to investigate the possible nonlinear correlations between the TyG index and clinical outcomes. Univariable and multivariable Cox proportional regression models were used to investigate the association between TyG index and prognosis. Hazard ratios (HRs) and 95% confidence intervals (CIs) were presented. The multivariable Cox models were adjusted for age, male sex, BMI, hypertension, diabetes mellitus, ACS presentation, MI histories, TC, LDL-C, hsCRP, serum creatinine, LVEF, and three-vessel disease. Moreover, the patients were stratified into 3 groups according to glycemic status to explore the impact of glucose metabolism on the association between the TyG index and prognosis in patients with bifurcation lesions. Subgroup analysis was performed to investigate the association between the TyG index and CV events differed by subgroup according to age, sex, BMI, hypertension presence, LDL-C and hsCRP levels, and the *P* value for interaction was determined by abovementioned multivariable Cox regression model. Improvements in risk discrimination of the TyG index beyond established clinical risk variables were assessed using C-statistics, the continuous net reclassification improvement (NRI), and the integrated discrimination improvement (IDI). Statistical significance was defined as a two-tailed *P* value < 0.05. R software version 4.1.2 (R Foundation for Statistical Computing, Vienna, Austria) was used for all statistical analyses.

## Results

### Baseline characteristics stratified by the occurrence of the primary endpoint

A total of 4530 patients with coronary bifurcation lesions were included in final analysis (Fig. 1). the distribution of the TyG index was depicted in additional file 1: Fig. S1. Baseline characteristics of patients with and without CV events are shown in Table 1. Patients experiencing CV events tended to be older with higher prevalence of DM, MI histories, PAD, CKD and three-vessel disease than those not (all *P* < 0.05). Besides, there were higher levels of SBP, FBG, HbA1c, hsCRP, serum creatinine and lower levels of BMI and LVEF in the events group than in the non-events group (all *P* < 0.05).

**Table 1:** Baseline characteristics stratified by the occurrence of the primary endpoint events

	Overall (n = 4530)	Non-events (n = 4389)	Events (n = 141)	*P* value
Age, years	59.53 ± 10.02	59.37 ± 9.95	64.63 ± 10.89	< 0.001
Male	3525 (77.8)	3417 (77.9)	108 (76.6)	0.802
BMI, kg/m^2^	25.89 ± 3.18	25.91 ± 3.18	25.27 ± 3.15	0.019
DM	1951 (43.1)	1877 (42.8)	74 (52.5)	0.027
Hypertension	2866 (63.3)	2768 (63.1)	98 (69.5)	0.141
SBP, mmHg	130.53 ± 17.66	130.38 ± 17.57	135.20 ± 19.85	0.001
DBP, mmHg	77.28 ± 10.93	77.32 ± 10.92	76.16 ± 11.43	0.217
Current smoker	1447 (31.9)	1406 (32.0)	41 (29.1)	0.516
ACS	2813 (62.1)	2734 (62.3)	79 (56.0)	0.155
Family history of CAD	562 (12.4)	544 (12.4)	18 (12.8)	0.998
Previous MI	1035 (22.8)	988 (22.5)	47 (33.3)	0.004
Previous PCI	896 (19.8)	859 (19.6)	37 (26.2)	0.064
Previous CABG	52 (1.1)	51 (1.2)	1 (0.7)	0.924
Previous stroke	582 (12.8)	557 (12.7)	25 (17.7)	0.103
Previous PAD	260 (5.7)	245 (5.6)	15 (10.6)	0.018
CKD	90 (2.0)	76 (1.7)	14 (9.9)	< 0.001
LVEF, %	62.56 ± 5.11	62.61 ± 4.97	60.98 ± 8.20	< 0.001
**Laboratory results**				
TyG	8.90 ± 0.57	8.89 ± 0.56	9.10 ± 0.66	< 0.001
FBG, mmol/L	6.46 ± 2.32	6.41 ± 2.24	7.81 ± 3.86	< 0.001
HbA1c, %	6.43 ± 1.21	6.41 ± 1.20	6.80 ± 1.47	< 0.001
TC, mmol/L	4.01 ± 1.03	4.00 ± 1.02	4.09 ± 1.12	0.323
TG, mmol/L	1.66 ± 0.82	1.66 ± 0.82	1.72 ± 0.80	0.177
LDL-C, mmol/L	2.41 ± 0.88	2.41 ± 0.88	2.49 ± 0.99	0.291
HDL-C, mmol/L	1.10 ± 0.30	1.10 ± 0.30	1.08 ± 0.36	0.356
hsCRP, mg/L	2.67 ± 3.07	2.65 ± 3.06	3.20 ± 3.33	0.006
Creatinine, µmol/L	82.92 ± 21.16	82.64 ± 20.80	91.43 ± 28.94	< 0.001
Lipoprotein(a), mg/L	30.42 ± 30.64	30.47 ± 30.66	28.92 ± 30.28	0.469
**Medications**				
Clopidogrel	3860 (85.2)	3736 (85.1)	124 (87.9)	0.419
ACEI/ARB	1170 (25.8)	1134 (25.8)	36 (25.5)	1.000
β-blocker	3994 (88.2)	3869 (88.2)	125 (88.7)	0.961
CCB	1634 (36.1)	1577 (35.9)	57 (40.4)	0.315
Statins	4383 (96.8)	4246 (96.7)	137 (97.2)	0.971
Antidiabetic drugs	1514 (33.4)	1455 (33.2)	59 (41.8)	0.039
Nitrate	4391 (96.9)	4257 (97.0)	134 (95.0)	0.281
**Angiographic findings**				
SYNTAX score	15.53 ± 5.76	15.51 ± 5.74	16.04 ± 6.54	0.507
Left main disease	532 (11.7)	512 (11.7)	20 (14.2)	0.434
three-vessel disease	1870 (41.3)	1799 (41.0)	71 (50.4)	0.033
Chronic total occlusion	392 (8.7)	374 (8.5)	18 (12.8)	0.107
Type B2/C lesion	3811 (84.1)	3690 (84.1)	121 (85.8)	0.660
Ostial lesions	1060 (23.4)	1029 (23.4)	31 (22.0)	0.763
Thrombotic lesions	59 (1.3)	57 (1.3)	2 (1.4)	1.000
Severe calcification	153 (3.4)	145 (3.3)	8 (5.7)	0.195

Values are presented as mean ± standard deviation or number (%). ACEI, angiotensin-converting enzyme inhibitor; ACS, acute coronary syndrome; ARB, angiotensin II receptor blocker; BMI, body mass index; CABG, coronary artery bypass grafting; CAD, coronary artery disease; CCB, calcium channel blocker; CKD, chronic kidney disease; DBP, diastolic blood pressure; DM, diabetes mellitus; FBG, fasting blood glucose; HbA1c, Hemoglobin A1c; HDL-C, high-density lipoprotein cholesterol; hsCRP, high sensitivity C-reactive protein; LDL-C, low-density lipoprotein cholesterol; LVEF, left ventricular ejection fraction; MI, myocardial infarction; PAD, peripheral artery disease; PCI, percutaneous coronary intervention; SBP, systolic blood pressure; SYNTAX, synergy between PCI with taxus and cardiac surgery; TC, total cholesterol; TG, triglyceride; TyG, triglyceride-glucose

### Baseline characteristics stratified by the TyG index tertiles

Compared to patients with low TyG index, those with elevated TyG index were younger, more likely to be female, and had lower BMI (Table 2). In the group with increased TyG index, comorbidities including DM, hypertension, and CKD were more common. Levels of FBG, HbA1c, TC, TG, LDL-C, hsCRP, and creatine were all greater in the higher TyG group compared to the lower TyG group. Patients with higher TyG index were more likely to have three-vessel disease according to the angiographic features. Additionally, we discovered that individuals with high TyG index had a higher proportion of beta-blockers and anti-diabetic medications (all *P* < 0.05, Table 2).

**Table 2:** Baseline characteristics according to tertiles of the TyG index

	T1 TyG < 8.633 (n = 1509)	T2 TyG [8.633,9.096) (n = 1509)	T3 TyG ≥ 9.096 (n = 1512)	P value
Age, years	60.26 ± 10.10	59.78 ± 10.08	58.57 ± 9.80	< 0.001
Male	1215 (80.5)	1169 (77.5)	1141 (75.5)	0.003
BMI, kg/m2	25.07 ± 3.12	26.04 ± 3.13	26.55 ± 3.10	< 0.001
DM	358 (23.7)	611 (40.5)	982 (64.9)	< 0.001
Hypertension	858 (56.9)	971 (64.3)	1037 (68.6)	< 0.001
SBP, mmHg	129.64 ± 17.78	130.99 ± 17.54	130.95 ± 17.64	0.058
DBP, mmHg	76.54 ± 10.71	77.43 ± 11.01	77.87 ± 11.04	0.003
Current smoker	467 (30.9)	471 (31.2)	509 (33.7)	0.210
ACS	904 (59.9)	965 (63.9)	944 (62.4)	0.069
Family history of CAD	176 (11.7)	184 (12.2)	202 (13.4)	0.351
Previous MI	335 (22.2)	354 (23.5)	346 (22.9)	0.712
Previous PCI	290 (19.2)	284 (18.8)	322 (21.3)	0.186
Previous CABG	20 (1.3)	18 (1.2)	14 (0.9)	0.576
Previous stroke	201 (13.3)	173 (11.5)	208 (13.8)	0.136
Previous PAD	103 (6.8)	77 (5.1)	80 (5.3)	0.083
CKD	18 (1.2)	29 (1.9)	43 (2.8)	0.005
LVEF, %	62.68 ± 4.90	62.41 ± 5.44	62.59 ± 4.97	0.338
**Laboratory results**				
TyG	8.30 ± 0.25	8.86 ± 0.14	9.53 ± 0.36	< 0.001
FBG, mmol/L	5.34 ± 0.95	6.07 ± 1.43	7.96 ± 3.09	< 0.001
HbA1c, %	5.96 ± 0.80	6.28 ± 0.97	7.03 ± 1.48	< 0.001
TC, mmol/L	3.68 ± 0.92	4.02 ± 0.98	4.32 ± 1.08	< 0.001
TG, mmol/L	0.99 ± 0.24	1.53 ± 0.34	2.45 ± 0.86	< 0.001
LDL-C, mmol/L	2.18 ± 0.80	2.48 ± 0.86	2.58 ± 0.94	< 0.001
HDL-C, mmol/L	1.21 ± 0.33	1.10 ± 0.28	1.01 ± 0.25	< 0.001
hsCRP, mg/L	2.32 ± 2.95	2.79 ± 3.21	2.89 ± 3.01	< 0.001
Creatinine, µmol/L	81.00 ± 15.77	83.04 ± 17.20	84.70 ± 28.13	< 0.001
Lipoprotein(a), mg/L	32.49 ± 31.10	30.35 ± 30.34	28.42 ± 30.37	0.001
**Medications**				
Clopidogrel	1298 (86.0)	1277 (84.6)	1285 (85.0)	0.536
ACEI/ARB	371 (24.6)	385 (25.5)	414 (27.4)	0.202
β-blocker	1288 (85.4)	1340 (88.8)	1366 (90.3)	< 0.001
CCB	511 (33.9)	547 (36.2)	576 (38.1)	0.052
Statins	1457 (96.6)	1456 (96.5)	1470 (97.2)	0.452
Antidiabetic drugs	308 (20.4)	460 (30.5)	746 (49.3)	< 0.001
Nitrate	1457 (96.6)	1459 (96.7)	1475 (97.6)	0.224
**Angiographic findings**				
SYNTAX score	15.29 ± 5.58	15.55 ± 5.78	15.74 ± 5.92	0.078
Left main disease	165 (10.9)	183 (12.1)	184 (12.2)	0.489
three-vessel disease	585 (38.8)	608 (40.3)	677 (44.8)	0.002
Chronic total occlusion	123 (8.2)	125 (8.3)	144 (9.5)	0.334
Type B2/C lesion	1269 (84.1)	1259 (83.4)	1283 (84.9)	0.564
Ostial lesions	363 (24.1)	357 (23.7)	340 (22.5)	0.571
Thrombotic lesions	15 (1.0)	18 (1.2)	26 (1.7)	0.192
Severe calcification	60 (4.0)	44 (2.9)	49 (3.2)	0.255

Values are presented as mean ± standard deviation or number (%) and comparison among groups was achieved by One-way ANOVA, Chi-square test or Fisher’s exact test as appropriate. Abbreviations as in Table 1

### Correlation between the TyG index and established risk factors

Pearson or spearman correlation analysis were used to assess the correlation between the TyG index and established risk factors (Table 3). The results showed that the TyG index was positively correlated with BMI, SBP, DBP, HbA1c, FBG, TC, TG, LDL-C, hsCRP and serum creatinine and negatively correlated with age, HDL-C and Lp(a) (all *P* < 0.05).

**Table 3:** Correlation between the TyG index and clinical risk factors

Variables	Correlation coefficient (r)	*P* value
Age, years	-0.078	< 0.001
BMI, kg/m^2^	0.202	< 0.001
SBP, mmHg	0.038	0.011
DBP, mmHg	0.055	< 0.001
HbA1c, %	0.422	< 0.001
FBG, mmol/L	0.588	< 0.001
TC, mmol/L	0.276	< 0.001
TG, mmol/L	0.825	< 0.001
LDL-C, mmol/L	0.187	< 0.001
HDL-C, mmol/L	-0.283	< 0.001
HSCRP, mg/L	0.082	< 0.001
Serum creatinine, µmol/L	0.106	< 0.001
Lipoprotein(a), mg/L	-0.053	< 0.001

Abbreviations as in Table 1

### Association between the TyG index and CV events

**Fig. 2:**
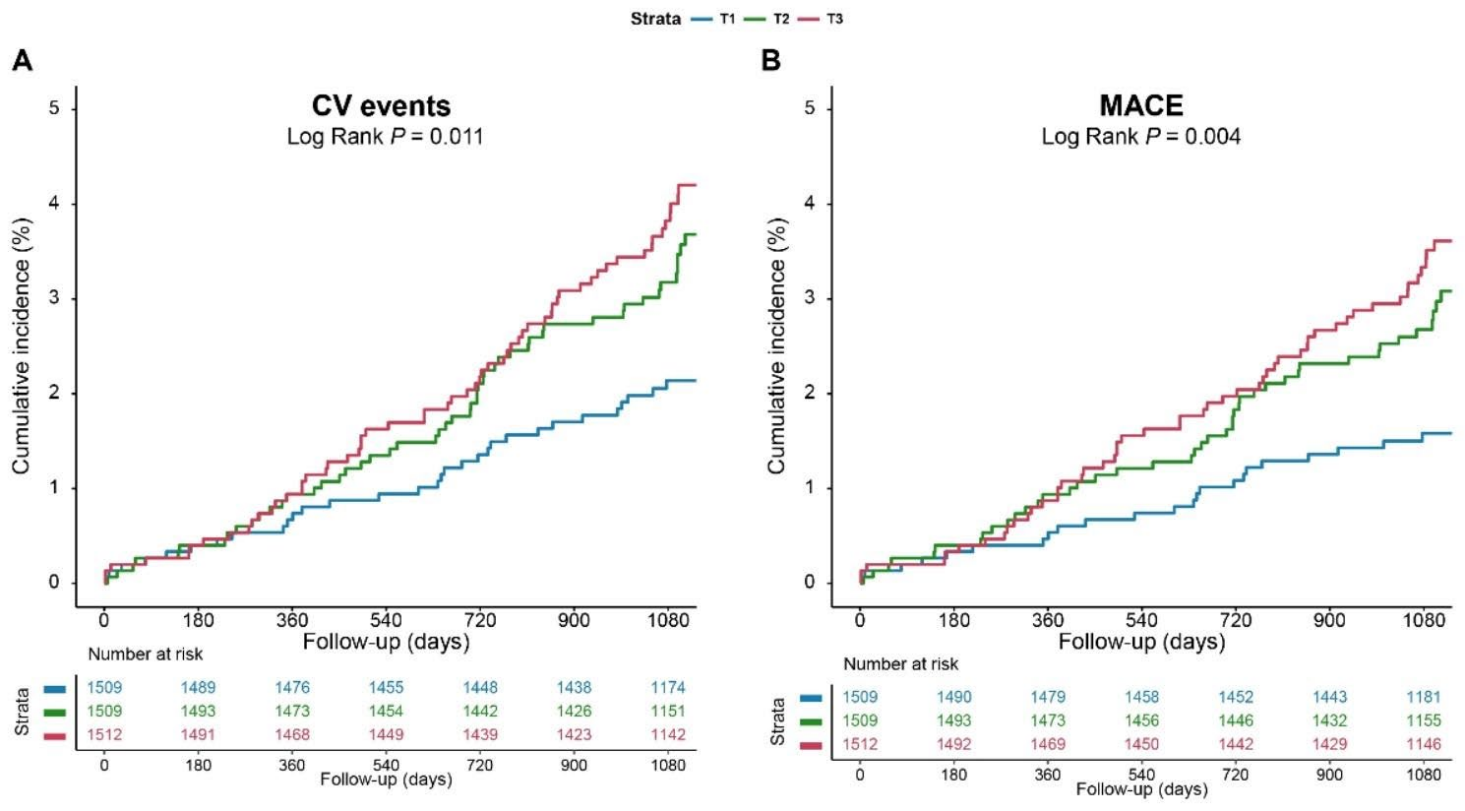
Kaplan-Meier curves for the TyG index and. CV events were defined as a composite of CV death, nonfatal MI, and non-fatal stroke. MACEs were defined as a composite of CV death and nonfatal MI. CV, cardiovascular; MACE, major adverse cardiac events; MI, myocardial infarction; TyG, triglyceride-glucose

During a median follow-up of 3.1 years, 141 (3.1%) CV events and 117 (2.6%) MACEs were recorded. RCS plots discovered linear relationships between the TyG index and CV events and MACEs (both non-linear *P* > 0.05; additional file 1: Fig. 2). As shown in Fig. 2, KM curves showed that the cumulative incidence of both CV events and MACEs increased incrementally across the TyG index tertiles (both Log rank *P* < 0.05).

Cox proportional regression analyses were implemented to evaluate the relationship between the TyG index and adverse clinical events (Table 4). In the univariable models, the risk of CV events (HR: 1.79, 95% CI: 1.37, 2.35; *P* < 0.001) or MACEs (HR: 1.85, 95% CI: 1.37, 2.49; *P* < 0.001) rose progressively with per 1-unit increase in the TyG index. And the associations remained significant after adjusting for age, sex, BMI, total cholesterol, LDL-C, hsCRP, creatine, LVEF, ACS, MI histories, hypertension, and three-vessel disease. When treated as a categorical variable, both second and highest tertiles of the TyG index (adjusted HR, 1.68; 95%CI, 1.06–2.65; *P* = 0.028; adjusted HR, 2.10; 95%CI, 1.28–3.47; *P* = 0.004, respectively) were associated with a higher risk of CV events compared to the low TyG group. A similar association was observed between the TyG index and MACEs.

**Table 4:** The TyG index in relation to CV events and MACEs

	Events (%)	Univariable model		Multivariable model*	
		HR (95%CI)	*P* value	HR (95%CI)	*P* value
**CV events** ^**a**^	141 (3.1)	1.79 (1.37–2.35)	< 0.001	2.14 (1.50–3.04)	< 0.001
T1	31 (2.1)	Reference	NA	Reference	NA
T2	51 (3.4)	1.65 (1.06–2.58)	0.027	1.68 (1.06–2.65)	0.028
T3	59 (3.9)	1.92 (1.24–2.96)	0.003	2.10 (1.28–3.47)	0.004
**MACEs** ^**b**^	117 (2.6)	1.85 (1.37–2.49)	< 0.001	1.94 (1.33–2.84)	< 0.001
T1	23 (1.5)	Reference	NA	Reference	NA
T2	43 (2.8)	1.88 (1.13–3.12)	0.015	1.87 (1.11–3.14)	0.019
T3	51 (3.4)	2.23 (1.37–3.65)	0.001	2.28 (1.30–4.01)	0.004

^a^CV events were defined as a composite of CV death, nonfatal MI, and non-fatal stroke. ^b^MACEs were defined as a composite of CV death and nonfatal MI. *Models adjusted for age, male sex, BMI, hypertension, diabetes mellitus, ACS presentation, histories of MI, TC, LDL-C, hsCRP, serum creatinine, LVEF, and three-vessel disease. CI, confidence interval; CV, cardiovascular; HR, hazard ratio; MACE, major adverse cardiac events; NA, not applicable. Other abbreviations as in Table 1

To investigate the extra predictive value of the TyG index, we built an original model including age, sex, BMI, total cholesterol, LDL-C, hsCRP, creatine, LVEF, ACS, MI histories, hypertension, and three-vessel disease and got a C-statistic of 0.678 (95%CI, 0.634–0.722) for CV events. The C-statistic was greatly enhanced by the addition of the TyG index, increasing to 0.696 (95%CI, 0.651–0.741) (ΔC-statistic, 0.018; *P* < 0.001; NRI, 0.23; *P* = 0.007; IDI, 0.81%, *P* = 0.004) (Table 5). As for MACEs, the addition of the TyG index improved the C-statistic from 0.700 (95%CI, 0.650–0.750) to 0.713 (95%CI, 0.664–0.763) (ΔC-statistic, 0.013; *P* < 0.001; NRI, 0.25; *P* = 0.003; IDI, 0.61%; *P* = 0.010).

**Table 5:** C-statistics of the TyG index for predicting CV events and MACEs in patients with bifurcation lesions

	C-statistic	ΔC-statistic	*P* value	NRI	*P* value	IDI	*P* value
CV events ^a^							
Original model*	0.678 (0.634–0.722)						
Original model + TyG index	0.696 (0.651–0.741)	0.018	< 0.001	0.23	0.007	0.81%	0.004
**MACEs** ^**b**^							
Original model	0.700 (0.650–0.750)						
Original model + TyG index	0.713 (0.664–0.763)	0.013	< 0.001	0.25	0.003	0.61%	0.010

^a^CV events were defined as a composite of CV death, nonfatal MI, and non-fatal stroke. ^b^MACEs were defined as a composite of CV death and nonfatal MI. ^*^Original model included age, male sex, BMI, hypertension, diabetes mellitus, ACS presentation, MI histories, TC, LDL-C, hsCRP, serum creatinine, LVEF, and three-vessel disease. Other abbreviation as in Table 1

### Subgroup analysis

**Fig. 3:**
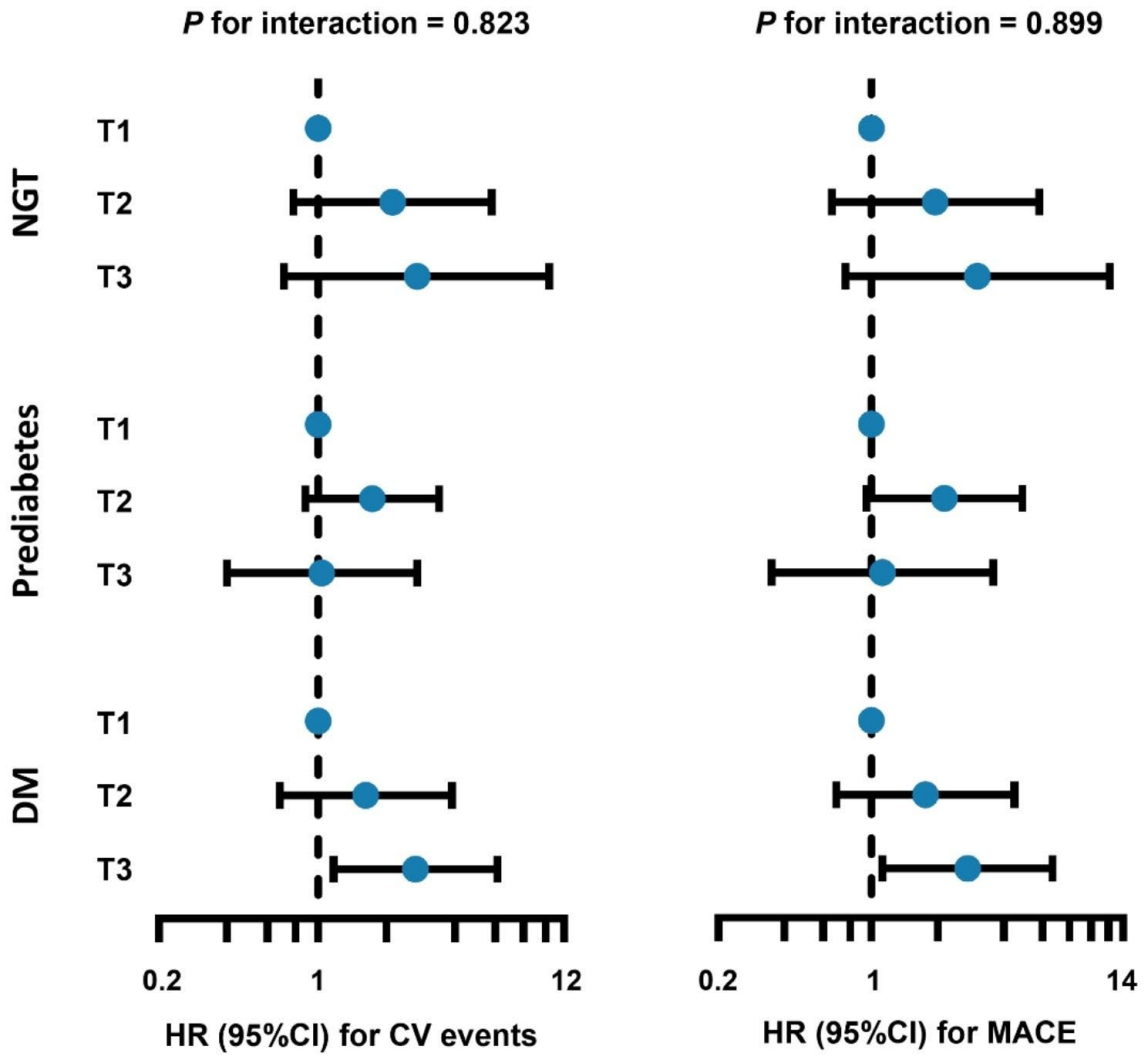
Forest plot for the TyG index and CV events or MACE according to different glycemic status. Models adjusted for age, male sex, BMI, hypertension, diabetes mellitus, ACS presentation, MI histories, TC, LDL-C, hsCRP, serum creatinine, LVEF, and three-vessel disease. Blue dots indicate HR value and Bars indicate 95%CIs. CI, confidence intervanl; CV, cardiovascular; DM, diabetes mellitus; HR, hazard ratio; MACE, major adverse cardiac event; NGT, normal glucose tolerance; TyG, triglyceride-glucose

As shown in Fig. 3 and additional file 1: Table S1, a higher TyG index was linked with a significantly increased risk of CV events whether the variable was considered as continuous (adjusted HR: 2.60, 95% CI: 1.69–4.02; *P* < 0.001) or categorical (T3 vs. T1, adjusted HR: 2.68, 95% CI: 1.17–6.11; *P* = 0.020) in patients with DM. whereas no significant association between the TyG index and CV events was observed in patients with NGT or prediabetes. The difference in the association between TyG index and CV events among patients with different glycemic statuses was not significant (*P* for interaction = 0.823). As for the secondary endpoint, diabetes patients with higher TyG index had a greater risk of MACE (adjusted HR: 2.21, 95% CI: 1.40, 3.48; *P* < 0.001). Furthermore, in the diabetes group, patients with high TyG index had a greater risk of MACE compared to patients with low TyG index (adjusted HR: 2.74, 95% CI: 1.12, 6.68; *P* = 0.899). The interaction effect of glycemic status and TyG index on MACE risk was not significant (*P* for interaction = 0.899).

**Fig. 4:**
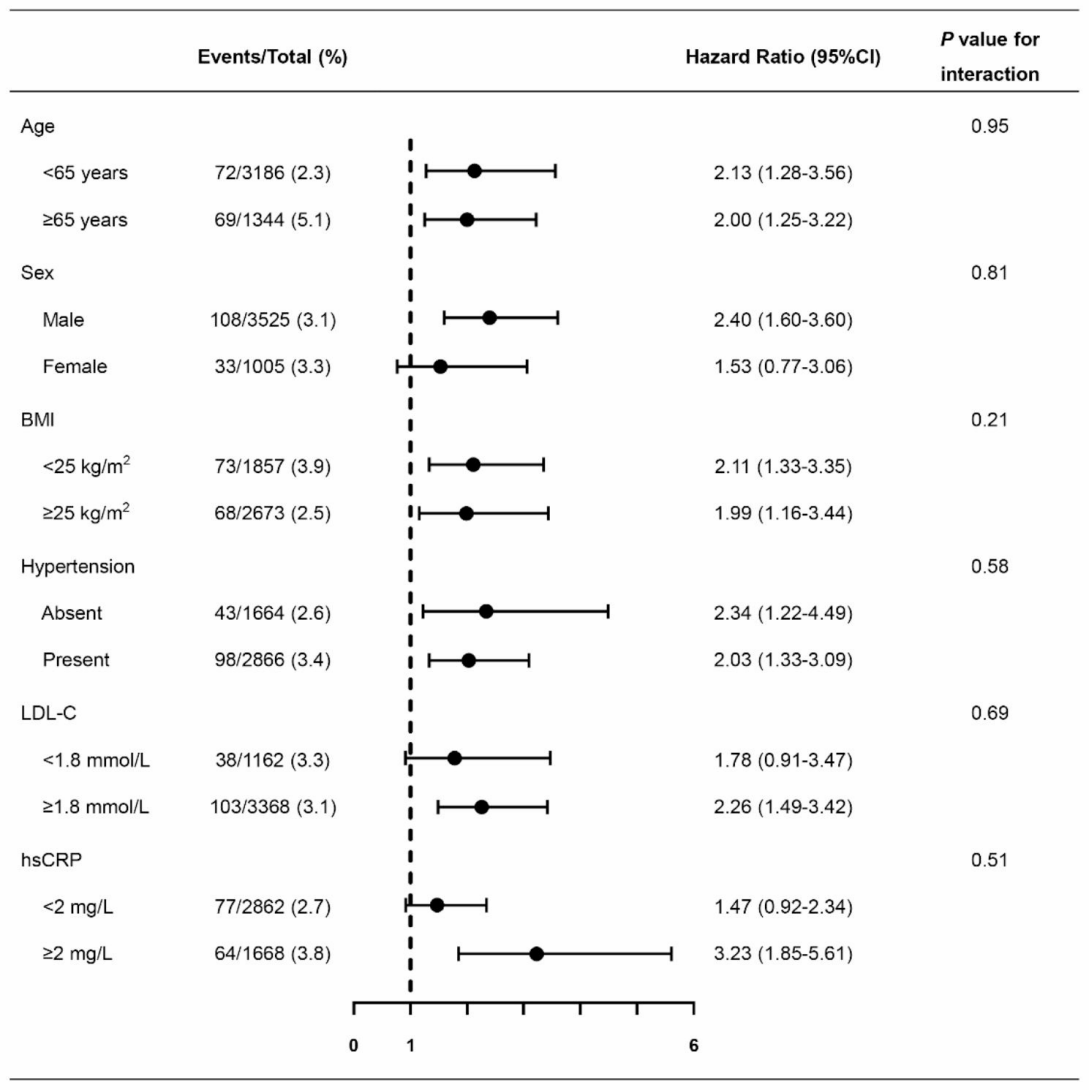
TyG index in relation to CV events across different subgroups. Models adjusted for age, male sex, BMI, hypertension, diabetes mellitus, ACS presentation, MI histories, TC, LDL-C, hsCRP, serum creatinine, LVEF, and three-vessel disease

Furthermore, elevated TyG index levels were consistently related to CV events in patients with bifurcation lesion across various subgroups (Fig. 4 and additional file 1: Table S2, all *P* for interaction > 0.05).

## Discussion

This large-scale prospective cohort study included 4530 angiography-proven CAD patients with bifurcation lesions and investigated the association between the TyG index and CV events at 3-year follow-up. Salient findings are as follows: [1] multivariable Cox proportional models suggested that higher TyG index (per 1-unit increase) was associated with 2.14-fold and 1.94-fold increased risk of CV events and MACEs, respectively, in patients with bifurcation lesions [2]. when patients were stratified into 3 groups (DM, prediabetes and NGT), increased TyG index levels were consistently associated with higher risk for both CV events and MACEs after adjusting for confounding factors (both *P* for interaction > 0.05). Specifically, higher TyG index (per 1-unit increase or the highest tertile) conferred significantly increased risk of CV events and MACEs at 3-year follow-up in diabetic patients [4]. the association between the TyG index and CV events at 3-year follow-up was consistent across various subgroups; [3] adding the TyG index to the model significantly improved the risk prediction for CV events and MACEs in patients with bifurcation lesions. Our findings demonstrated, for the first time, that an increased TyG index was associated with poor prognosis in patients with bifurcation lesions, suggesting the TyG index could help in risk stratification in this population.

Increasing evidence have demonstrated that an increased TyG index was related to poor prognosis in patients with CAD. A previous nested case–control study by Jin et al. [15] revealed that an elevated TyG index was associated with higher risk of major adverse cardiovascular and cerebrovascular events (MACCEs) after adjusting for confounding factors among 1282 diabetic patients with stable CAD. Besides, this study suggested that adding the TyG index to a Cox regression model including HbA1c could significantly increase the predictive performance for MACCEs [15]. As for CAD patients with different glycemic status, previous studies by Yang et al. [16] and Si et al. [17] demonstrated respectively that an increased TyG index was associated with poor prognosis in nondiabetic patients after percutaneous coronary intervention and in diabetic patients with a middle age and male sex. In addition, previous studies have also demonstrated that the TyG index could be a promising marker for risk stratification and prognosis for ACS patients [18–20]. A cohort study including 2531 patients with ACS and established DM showed that the occurrence of 3-year MACEs increased with TyG index tertiles and the TyG index was an independent predictor for MACEs after adjustment for CV risk factors and invasive treatments [19]. Furthermore, studies conducted by Luo et al. [18] and Zhang et al. [20] similarly showed the association between the TyG index and prognosis in patients with acute ST-elevation myocardial infarction after PCI and diabetic patients with acute MI, respectively.

Recently, studies by Mao et al. [21] and Wang et al. [22] illustrated that the TyG index was independently associated with higher SYNTAX score (OR, 6.055; 95%CI, 2.915–12.579) and the incidence of multi-vessel disease (OR, 1.355; 95%CI, 1.154–1.591), respectively. Besides, a large-scale observational study determined that elevated TyG index was correlated with the occurrence of impaired collateralization (ORs, 1.59 and 5.72 in the second and third tertile groups than in the first tertile group) in patients with CTO lesions [23]. Collectively, these findings provided incremental information that elevated TyG index was related to increased coronary lesion complexity. For clinical outcomes, Song et al. [8] enrolled 2740 patients with CTO lesions and showed a significant relationship between the TyG index and MACCEs at 3-year follow-up, which further confirmed the prognostic value of the TyG index in patients with complex coronary lesions. Coronary bifurcation lesions, as one of complex coronary lesion subsets, conferred a higher risk for adverse clinical events than those non-bifurcation lesions [3, 24]. Accordingly, it is crucial to identify those patients with bifurcation lesions who are at increased risk for CV events where more therapeutics can be provided. Here, we demonstrated, for the first time, that the TyG index was positively associated with the incidence of CV events or MACEs at long-term follow-up in patients with bifurcation lesions. Moreover, addition of the TyG index could significantly improve the prognostication of the original model, which delivered novel information that the TyG index is a promising marker for risk stratification and prognosis in this population. Additionally, previous studies have also suggested that atherogenic index of plasma (AIP), an indication of atherogenic lipoprotein status, was strongly associated with CV events in both diabetic and nondiabetic patients [25]. Increased HDL-C levels within the normal range suggests there is an opposing effect of TG on atherosclerosis, with lower HDL-C levels appearing to inhibit anti-atherogenic properties and the anti-oxidation effect. These effects appear to be attributed to diminishing HDL-C levels which are usually observed prior to the presence of glycemic dysregulation [26]. Previous research has demonstrated that higher TyG did not accurately reflect a decrease in HDL-C levels and the TyG index might not a suitable prognostic marker for nondiabetic patients where the AIP could be useful [16, 27]. Further studies are warranted to compare the predictive performance among these markers.

The adoption of the TyG index as a prognostic marker in CAD patients might be influenced by diabetic and hyperlipidemic state that led to CAD [6]. Previously, a retrospective cohort study assessed the relationship between the TyG index and lesion severity in CAD patients according to distinct glucose metabolism. The results showed that the association between TyG index and the occurrence of multi-vessel lesions was more significant in diabetic patients, achieving the highest OR among the different glycemic status (OR, 1.717; 95%CI, 1.161–2.539) [9]. Besides, consistent data illustrated that elevated TyG index could predict poor prognosis in CAD patients with DM [19, 20, 28, 29]. however, previous studies showed that the TyG index presumably not an effective predictor for CV events in nondiabetic patients undergoing PCI [16] or with ACS and LDL-C levels below 1.8 mmol/L [30]. Nonetheless an increased TyG index might be a useful predictor of subsequent revascularization among nondiabetic patient with ACS [30]. Similar to findings from previous studies, we demonstrated that the prognostic value of the TyG index for CV events or MACEs were more obvious in patients with bifurcation lesions with DM but not prediabetes or NGT. Furthermore, we also found that the association between the TyG index and CV events were consistent in different subgroups (such as LDL-C and hsCRP), indicating that the TyG index could be a prognostic marker in different metabolic status.

There are several limitations in this study. First, this was a single-center study only included Chinese patients with bifurcation lesions, which possibly influenced the applicability of our findings to other populations [31]. Second, due to the nature of observational study design, potential confounding factors could not be fully eradicated [32]. Third, follow-up data on the TyG index was unavailable, which may have clinical significance. Fourth, insulin levels were not measured in patients included in this study, and HOMA-IR values could not be calculated. Further prospective studies with long-term follow-up are warranted to confirm our findings. Fifth, information regarding other medications such as fibrates was not available. Further prospective studies are warranted to confirm our findings.

## Conclusions

High TyG index was associated with CV events in patients with bifurcation lesions, suggesting the TyG index could help in risk stratification in this population.

### Electronic supplementary material

Below is the link to the electronic supplementary material.

Supplementary Material 1

## Data Availability

The datasets used and/or analysed during the current study are available from the corresponding author on reasonable request.

## References

[1] Dou K, Zhang D, Xu B (2015). An angiographic tool for risk prediction of side branch occlusion in coronary bifurcation intervention: the RESOLVE score system (risk prEdiction of side branch OccLusion in coronary bifurcation interVEntion). JACC Cardiovasc Interv.

[2] Levine GN, Bates ER, Blankenship JC (2011). 2011 ACCF/AHA/SCAI Guideline for Percutaneous Coronary intervention: a report of the American College of Cardiology Foundation/American Heart Association Task Force on Practice Guidelines and the Society for Cardiovascular Angiography and interventions. Circulation.

[3] Ninomiya K, Serruys PW, Garg S (2022). Predicted and observed mortality at 10 years in patients with bifurcation lesions in the SYNTAX trial. JACC Cardiovasc Interv.

[4] Pan M, Lassen JF, Burzotta F (2023). The 17th expert consensus document of the European Bifurcation Club - techniques to preserve access to the side branch during stepwise provisional stenting. EuroIntervention.

[5] Mauri L, Hsieh W-h, Massaro JM (2007). Stent Thrombosis in randomized clinical trials of drug-eluting stents. N Engl J Med.

[6] Tao L-C, Xu J-N, Wang T-T (2022). Triglyceride-glucose index as a marker in Cardiovascular Diseases: landscape and limitations. Cardiovasc Diabetol.

[7] Minh HV, Tien HA, Sinh CT (2021). Assessment of preferred methods to measure insulin resistance in Asian patients with Hypertension. J Clin Hypertens (Greenwich).

[8] Song Y, Cui K, Yang M (2023). High triglyceride-glucose index and stress hyperglycemia ratio as predictors of adverse cardiac events in patients with coronary chronic total occlusion: a large-scale prospective cohort study. Cardiovasc Diabetol.

[9] Su J, Li Z, Huang M (2022). Triglyceride glucose index for the detection of the severity of coronary artery Disease in different glucose metabolic states in patients with coronary Heart Disease: a RCSCD-TCM study in China. Cardiovasc Diabetol.

[10] Ding X, Wang X, Wu J (2021). Triglyceride-glucose index and the incidence of atherosclerotic Cardiovascular Diseases: a meta-analysis of cohort studies. Cardiovasc Diabetol.

[11] Lunardi M, Louvard Y, Lefèvre T (2022). Definitions and standardized endpoints for treatment of coronary bifurcations. J Am Coll Cardiol.

[12] Chen SL, Sheiban I, Xu B (2014). Impact of the complexity of bifurcation lesions treated with drug-eluting stents: the DEFINITION study (definitions and impact of complEx biFurcation lesIons on clinical outcomes after percutaNeous coronary IntervenTIOn using drug-eluting steNts). JACC Cardiovasc Interv.

[13] He J, Song C, Wang H et al. Diabetes Mellitus with mild or moderate kidney dysfunction is Associated with poor prognosis in patients with coronary artery Disease: a large-scale cohort study. Diabetes Res Clin Pract. 2023:110693. 10.1016/j.diabres.2023.110693 37160234

[14] He J, Lin Z, Song C (2023). High absolute neutrophil count with type 2 Diabetes is associated with adverse outcome in patients with coronary artery Disease: a large-scale cohort study. Front Endocrinol (Lausanne).

[15] Jin J-L, Sun D, Cao Y-X (2018). Triglyceride glucose and haemoglobin glycation index for predicting outcomes in Diabetes patients with new-onset, stable coronary artery Disease: a nested case-control study. Ann Med.

[16] Yang J, Tang Y-D, Zheng Y (2021). The impact of the triglyceride-glucose index on poor prognosis in NonDiabetic patients undergoing percutaneous coronary intervention. Front Endocrinol (Lausanne).

[17] Si Y, Fan W, Shan W (2021). Association between triglyceride glucose index and coronary artery Disease with type 2 Diabetes Mellitus in middle-aged and elderly people. Medicine.

[18] Luo E, Wang D, Yan G (2019). High triglyceride-glucose index is associated with poor prognosis in patients with acute ST-elevation Myocardial Infarction after percutaneous coronary intervention. Cardiovasc Diabetol.

[19] Wang L, Cong H-L, Zhang J-X (2020). Triglyceride-glucose index predicts adverse cardiovascular events in patients with Diabetes and acute coronary syndrome. Cardiovasc Diabetol.

[20] Zhang Y, Ding X, Hua B (2021). Predictive effect of triglyceride–glucose index on clinical events in patients with type 2 Diabetes Mellitus and acute Myocardial Infarction: results from an observational cohort study in China. Cardiovasc Diabetol.

[21] Mao Q, Zhou D, Li Y (2019). The triglyceride-glucose index predicts coronary artery Disease Severity and Cardiovascular outcomes in patients with Non-ST-Segment elevation Acute Coronary Syndrome. Dis Markers.

[22] Wang X, Xu W, Song Q (2022). Association between the triglyceride-glucose index and severity of coronary artery Disease. Cardiovasc Diabetol.

[23] Gao A, Liu J, Hu C (2021). Association between the triglyceride glucose index and coronary collateralization in coronary artery Disease patients with chronic total occlusion lesions. Lipids Health Dis.

[24] Girasis C, Farooq V, Diletti R (2013). Impact of 3-dimensional bifurcation angle on 5-year outcome of patients after percutaneous coronary intervention for left main coronary artery Disease: a substudy of the SYNTAX trial (synergy between percutaneous coronary intervention with taxus and cardiac Surgery). JACC Cardiovasc Interv.

[25] Askina L, Tanriverdib O (2023). Is the Atherogenic Index of Plasma (AIP) a Cardiovascular Disease marker?. Cor Vasa.

[26] Denimal D, Nguyen A, Pais de Barros J-P (2016). Major changes in the sphingophospholipidome of HDL in non-diabetic patients with metabolic syndrome. Atherosclerosis.

[27] Zheng Y, Li C, Yang J (2022). Atherogenic index of plasma for non-diabetic, coronary artery Disease patients after percutaneous coronary intervention: a prospective study of the long-term outcomes in China. Cardiovasc Diabetol.

[28] Zhao Q, Zhang T-Y, Cheng Y-J (2020). Impacts of triglyceride-glucose index on prognosis of patients with type 2 Diabetes Mellitus and non-ST-segment elevation acute coronary syndrome: results from an observational cohort study in China. Cardiovasc Diabetol.

[29] Tai S, Fu L, Zhang N (2022). Association of the cumulative triglyceride-glucose index with major adverse cardiovascular events in patients with type 2 Diabetes. Cardiovasc Diabetol.

[30] Zhang Y, Ding X, Hua B (2022). High triglyceride-glucose index is Associated with Poor Cardiovascular outcomes in nondiabetic patients with ACS with LDL-C below 1.8 mmol/L. J Atheroscler Thromb.

[31] He J, Yang M, Song C et al. Lipoprotein(a) is associated with recurrent cardiovascular events in patients with coronary artery Disease and prediabetes or Diabetes. J Endocrinol Invest. 2023. 10.1007/s40618-023-02203-3 37777699

[32] He J, Bian X, Song C (2022). High neutrophil to lymphocyte ratio with type 2 Diabetes Mellitus predicts poor prognosis in patients undergoing percutaneous coronary intervention: a large-scale cohort study. Cardiovasc Diabetol.

