# Supplementary material for: High triglyceride-glucose index predicts cardiovascular events in patients with coronary bifurcation lesions: a large-scale cohort study
Source: Cardiovasc Diabetol. 2023 Oct 27;22:289. doi: 10.1186/s12933-023-02016-x (PMC10612152; doi:10.1186/s12933-023-02016-x)
Supplement: Supplementary file 1 — Supplementary Material 1 [file 12933_2023_2016_MOESM1_ESM.docx]

**High triglyceride-glucose index predicts cardiovascular events in patients with coronary bifurcation lesions: a large-scale cohort study**

**Additional file 1**

**Contents**

[Table S1 Associations between the TyG index and CV events or MACEs according to glycemic status 2](#_Toc144816313)

[Table S2 Associations between the TyG index and CV events according to different subgroups 3](#_Toc144816314)

[Fig. S1 histogram of the TyG index 5](#_Toc144816315)

[Fig. S2 restrict cubic spine 6](#_Toc144816316)

# Table S1 Associations between the TyG index and CV events or MACEs according to glycemic status

|  | **Events (%)** | **Univariable model** | | **Multivariable model*** | |
| --- | --- | --- | --- | --- | --- |
|  |  | **HR (95%CI)** | ***P* value** | **HR (95%CI)** | ***P* value** |
| **CV events^a^** |  |  |  |  |  |
| NGT | 23 (2.5) | 2.13 (0.91-4.99) | 0.082 | 2.51 (0.82-7.69) | 0.108 |
| T1 | 8 (1.6) | Reference | NA | Reference | NA |
| T2 | 10 (3.3) | 2.04 (0.80-5.16) | 0.134 | 2.12 (0.78-5.77) | 0.140 |
| T3 | 5 (4.0) | 2.39 (0.78-7.30) | 0.127 | 2.72 (0.71-10.34) | 0.143 |
| Prediabetes | 44 (2.6) | 0.77 (0.41-1.46) | 0.424 | 1.06 (0.51-2.22) | 0.877 |
| T1 | 16 (2.4) | Reference | NA | Reference | NA |
| T2 | 21 (3.5) | 1.47 (0.77-2.82) | 0.246 | 1.73 (0.88-3.41) | 0.111 |
| T3 | 7 (1.7) | 0.72 (0.30-1.75) | 0.468 | 1.04 (0.40-2.73) | 0.932 |
| DM | 74 (3.8) | 2.17 (1.50-3.14) | <0.001 | 2.60 (1.69-4.02) | <0.001 |
| T1 | 7 (2.0) | Reference | NA | Reference | NA |
| T2 | 20 (3.3) | 1.68 (0.71-3.97) | 0.239 | 1.62 (0.68-3.87) | 0.273 |
| T3 | 47 (4.8) | 2.48 (1.12-5.48) | 0.025 | 2.68 (1.17-6.11) | 0.020 |
| P for interaction |  |  | 0.484 |  | 0.823 |
| **MACE^b^** |  |  |  |  |  |
| NGT | 20 (2.2) | 2.24 (0.90-5.58) | 0.082 | 2.49 (0.75-8.23) | 0.136 |
| T1 | 7 (1.4) | Reference | NA | Reference | NA |
| T2 | 8 (2.6) | 1.86 (0.67-5.13) | 0.231 | 1.95 (0.66-5.82) | 0.229 |
| T3 | 5 (4.0) | 2.73 (0.87-8.60) | 0.087 | 3.04 (0.76-12.16) | 0.116 |
| Prediabetes | 32 (1.9) | 0.87 (0.41-1.83) | 0.714 | 1.09 (0.46-2.61) | 0.842 |
| T1 | 10 (1.5) | Reference | NA | Reference | NA |
| T2 | 17 (2.9) | 1.91 (0.87-4.16) | 0.106 | 2.15 (0.95-4.86) | 0.067 |
| T3 | 5 (1.2) | 0.83 (0.28-2.41) | 0.726 | 1.12 (0.35-3.58) | 0.843 |
| DM | 65 (3.3) | 1.98 (1.33-2.94) | <0.001 | 2.21 (1.40-3.48) | <0.001 |
| T1 | 6 (1.7) | Reference | NA | Reference | NA |
| T2 | 18 (2.9) | 1.76 (0.70-4.43) | 0.231 | 1.76 (0.69-4.46) | 0.236 |
| T3 | 41 (4.2) | 2.52 (1.07-5.93) | 0.035 | 2.74 (1.12-6.68) | 0.027 |
| P for interaction |  |  | 0.570 |  | 0.899 |

^a^CV events were defined as a composite of CV death, nonfatal MI and nonfatal stroke

^b^MACE was defined as a composite of CV death, and nonfatal MI

*Models adjusted for age, male sex, BMI, hypertension, diabetes mellitus, ACS presentation, histories of MI, TC, LDL-C, hsCRP, serum creatinine, LVEF, and three-vessel disease.

ACS, acute coronary syndrome; BMI, body mass index; CI, confidence interval; CV, cardiovascular; DM, diabetes mellitus; HR, hazard ratio; hsCRP, high sensitivity C-reactive protein; LDL-C, low-density lipoprotein cholesterol; LVEF, left ventricular ejection fraction; MACE, major adverse cardiac events; NGT, normal glucose tolerance; MI, myocardial infarction; NA, not applicable; TC, total cholesterol; TyG triglyceride-glucose.

# Table S2 Associations between the TyG index and CV events according to different subgroups

| Variables | Events (%) | Unadjusted HR (95%CI) | *P* value | *P* for interaction | Adjusted HR (95%CI)* | *P* value | *P* for interaction |
| --- | --- | --- | --- | --- | --- | --- | --- |
| **Age, yrs** |  |  |  | 0.961 |  |  | 0.947 |
| <65 | 72/3186 | 1.87 (1.28-2.74) | 0.001 |  | 2.13 (1.28-3.56) | 0.004 |  |
| ≥65 | 69/1344 | 1.90 (1.29-2.81) | 0.001 |  | 2.00 (1.25-3.22) | 0.004 |  |
| **Sex** |  |  |  | 0.898 |  |  | 0.810 |
| Male | 108/3525 | 1.77 (1.30-2.42) | <0.001 |  | 2.40 (1.60-3.60) | <0.001 |  |
| Female | 33/1005 | 1.85 (1.05-3.25) | 0.032 |  | 1.53 (0.77-3.06) | 0.226 |  |
| **BMI, kg/m^2^** |  |  |  | 0.161 |  |  | 0.211 |
| <25 | 73/1857 | 2.29 (1.60-3.27) | <0.001 |  | 2.11 (1.33-3.35) | 0.002 |  |
| ≥25 | 68/2673 | 1.54 (1.02-2.33) | 0.038 |  | 1.99 (1.16-3.44) | 0.013 |  |
| **Hypertension** |  |  |  | 0.726 |  |  | 0.582 |
| Absent | 43/1664 | 1.90 (1.16-3.12) | 0.011 |  | 2.34 (1.22-4.49) | 0.010 |  |
| Present | 98/2866 | 1.71 (1.23-2.38) | 0.001 |  | 2.03 (1.33- 3.09) | 0.001 |  |
| **LDL-C, mmol/L** |  |  |  | 0.287 |  |  | 0.690 |
| <1.8 | 38/1162 | 1.44 (0.87-2.40) | 0.159 |  | 1.78 (0.91-3.47) | 0.093 |  |
| ≥1.8 | 103/3368 | 2.00 (1.45-2.76) | <0.001 |  | 2.26 (1.49-3.42) | <0.001 |  |
| **hsCRP, mg/L** |  |  |  | 0.562 |  |  | 0.507 |
| <2 | 77/2862 | 1.61 (1.09-2.37) | 0.017 |  | 1.47 (0.92-2.34) | 0.107 |  |
| ≥2 | 64/1668 | 1.89 (1.28-2.78) | 0.001 |  | 3.23 (1.85-5.61) | <0.001 |  |

*Models adjusted for age, male sex, BMI, hypertension, diabetes mellitus, ACS presentation, histories of MI, TC, LDL-C, hsCRP, serum creatinine, LVEF, and three-vessel disease.

Abbreviations as in Table S1.

# Fig. S1 histogram of the TyG index

**
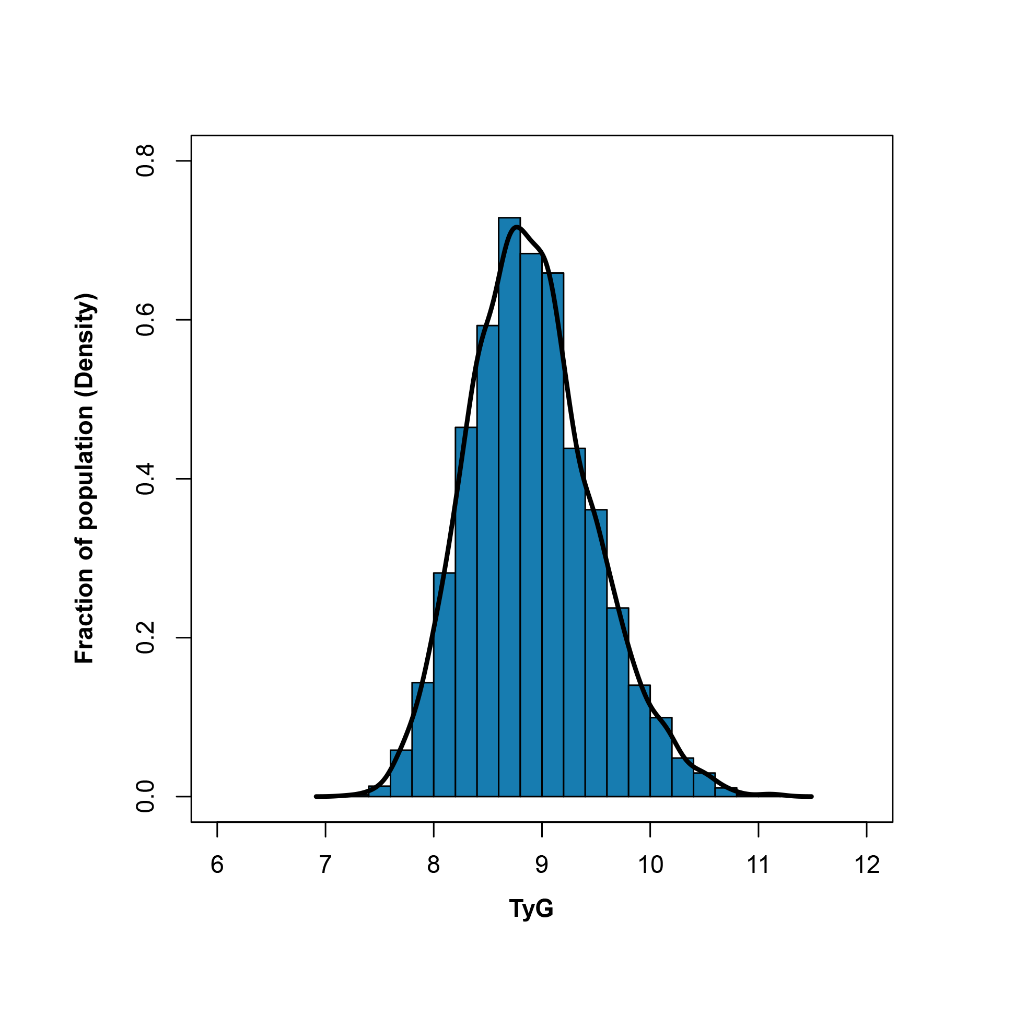
**

# Fig. S2 restrict cubic spine analysis between the TyG index and primary or secondary endpoints

**
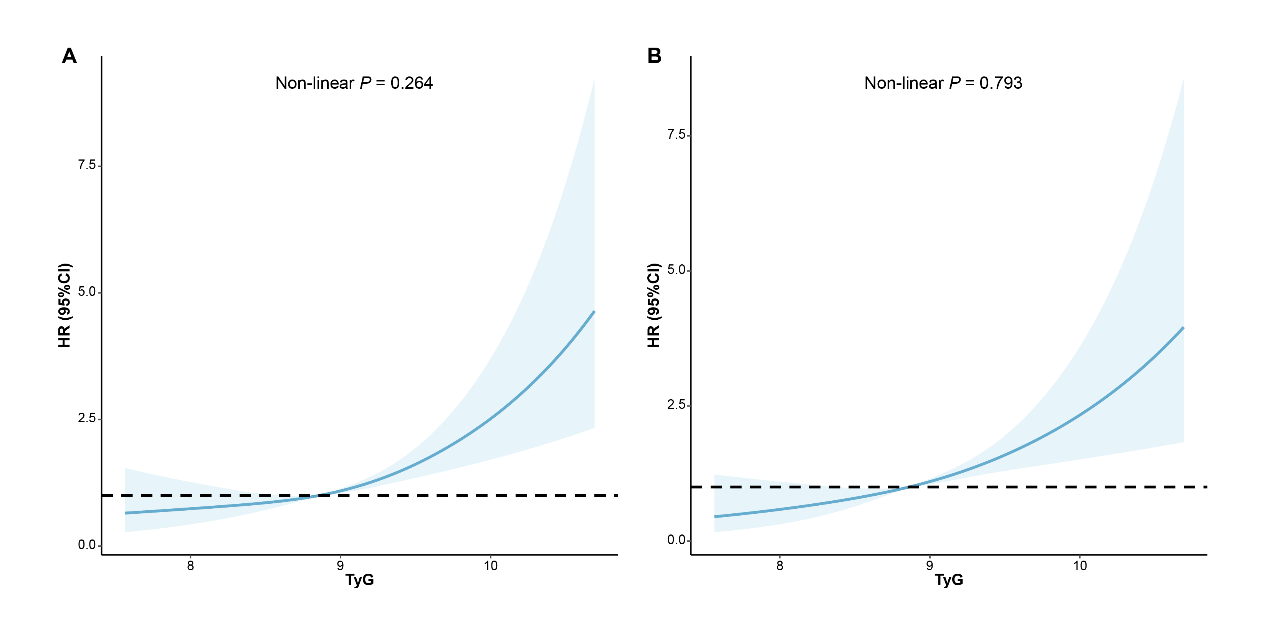
**

Models adjusted for age and male sex.

Abbreviations as in Table S1.
